# Supplementary material for: Factors affecting caregivers’ HPV vaccination decisions for adolescent girls: A secondary analysis of a Chinese RCT
Source: PLoS One. 2025 Jun 17;20(6):e0324260. doi: 10.1371/journal.pone.0324260 (PMC12173375; doi:10.1371/journal.pone.0324260)
Supplement: S1 File — (DOCX) [file pone.0324260.s001.docx]

**S1 File:** **Supplementary materials used in primary study**

**Questionnaire for the HPV Vaccination Promotion Project for Adolescent Girls aged 15-18 years (Pay-it-forward arm)**

ID number:

Adolescent girl’s name:

**Part A: Basic Information**

1. Your gender:

A. Male

B. Female

2. Your ethnicity:

A. Han

B. Others, please specify: __________

3. Your birth date __________ (e.g. "1990.01.01")

4. Your marital status is

A. Unmarried

B. Engaged or married

C. Separated or divorced

D. Widowed

E. Others, please specify: __________

5. The kinship between you and your child is

A. Father

B. Mother

C. Grandfather or grandfather

D. Grandmother or grandmother

E. Others, please specify: __________

6. Your highest education level is

A. Primary school or below

B. Junior high school

C. High school

D. Bachelor's degree or college

E. Postgraduate or above

7. Your current occupation is

A. Civil servant

B. Farmer

C. Ordinary worker (blue collar or labor-intensive work)

D. Company employee (white collar or office work)

E. Technician

F. Unemployed or retired

G. Others, please specify: __________

8. The annual income of your family is

A. 0-10,000 CNY/year (0-1,415 USD/Year)

B. 10000-30000 CNY /year (1,415-4,354 USD/Year)

C. 30,000-80000 CNY /year (4,354-11,611USD/Year)

D. 80000-150000 CNY /year (11,611-21,771USD/Year)

E. 150,000-300,000 CNY /year (21771-43541USD/Year)

F. 300000-1000000 CNY /year (43,541-145,137USD/Year)

G. More than 1,000,000 CNY /year (145,137 USD /Year or above)

9. Whether anyone in the same household has ever been infected with HPV?

A. Yes

B. No

C. No clear

10. Whether anyone in the same household has ever been infected with cancer cervical?

A. Yes

B. No

C. No clear

**Part B: Intention to receive vaccination**

11. Today researchers introduced you to the HPV vaccine and the "Pay-it-forward" program, would you like your child to participate in the receive the HPV vaccine?

A. Yes

B. No (please skip to question 13)

12. If you would like to have your child vaccinated, what are the reasons? [Multiple choice]

A. Have a plan for HPV vaccination

B. It will protect my child from cervical cancer

C. Recommendation from a friend/family member

D. Advice from a medical professional (not related to the Pay-it-forward project)

E. "Pay-it-forward" project

13. If you do not want your child to be vaccinated, what are the reasons? (If "Yes" is selected for question 11, skip this question) [Multiple choice]

A. Don't know enough about cervical cancer and HPV vaccine

B. Unsure of the effectiveness of the HPV vaccine

C. Family members do not allow vaccination

D. Too much trouble

E. Worried about side effects

F. Price reason

G. Others, please specify: _______

14. Would you like to donate some money to support other girls in our community to receive HPV vaccination?

A. Yes

B. No

15. How much would you be willing to donate for the next girl? (Skip this question if you answered "No" to question 14)

A. 330 CNY (to help the next girl get about one dose of the bivalent domestic HPV vaccine)

B. 165 CNY (to help cover about half of one dose of bivalent domestic HPV vaccine for the next girl)

C. 110 CNY (to help the next girl cover about 1/3 of a single dose of the bivalent domestic HPV vaccine)

D. Another amount, please specify:

16. What do you think are the benefits of the "Pay-it-forward" project? [Multiple choice]

A. Reducing my financial burden

B. I learned that HPV vaccine can prevent cervical cancer

C. More girls can be vaccinated against cervical cancer

D. It will reduce the spread of HPV infection

E. Spread love and warmth in the community

F. Other, please specify:

**Part C: Vaccine-related information**

17. Have you ever heard about the HPV prior to this program?

A. Yes

B. No

18. Have you ever heard about HPV vaccines prior to this program?

A. Yes

B. No

19. Have ever heard the piloting projects to cover the HPV vaccination of 13-14 years old girls in Chengdu at the end of 2021?

A. Yes

B. No

20. Were you aware of the following vaccines marketed in this country prior to this program

|  | A. Yes | B. No |
| --- | --- | --- |
| 9v |  |  |
| 4v |  |  |
| 2v* |  |  |

*2v: both imported and domestic (same applies to later questions)

21. In general, I think HPV vaccine is important.

|  | A strongly disagree | B disagree | C agree | D strongly agree |
| --- | --- | --- | --- | --- |
| 9v |  |  |  |  |
| 4v |  |  |  |  |
| 2v |  |  |  |  |

22. In general, I think the HPV vaccine is safe.

|  | A strongly disagree | B disagree | C agree | D strongly agree |
| --- | --- | --- | --- | --- |
| 9v |  |  |  |  |
| 4v |  |  |  |  |
| 2v |  |  |  |  |

23. In general, I think the HPV vaccine is effective.

|  | A strongly disagree | B disagree | C agree | D strongly agree |
| --- | --- | --- | --- | --- |
| 9v |  |  |  |  |
| 4v |  |  |  |  |
| 2v |  |  |  |  |

24. Has anyone around you been vaccinated against HPV?

A. Yes

B. No

25. Have you ever been hesitant about getting the HPV vaccination (except for allergies)?

A. Yes

B. No

26. Have you ever postponed the HPV vaccination (except for allergies)?

A. Yes

B. No

27. Have you ever refused to receive the HPV vaccination (except for allergies)?

A. Yes

B. No

28. Have you ever heard about negative information about HPV vaccination?

A. Yes

B. No

29. Do any of your friends or relatives object to HPV vaccination?

A. Yes

B. No

30. Have you or people around you had experienced adverse effects associated with HPV vaccination?

A. Yes

B. No

31. Is the price of the vaccine a barrier to HPV vaccination for you or others around you?

A. Yes

B. No

32. Do you think that, after HPV vaccination, your child still needs regular cervical cancer screening after they get married/become sexually active?

A. Yes

B. No

33. Which of the above three HPV vaccines would you preferably want your daughter to receive?

A. 2v-HPV vaccines (domestic or imported), because ________ (answer to "36")

B. Imported 4v-HPV vaccines, because ________ (answer to question "34")

C. Imported 9v-HPV vaccines, because ________ (answer to question "34")

D. No preferences, all of the above are fine (answer "36")

E. Not sure (answer "36")

34. If your community health center has no supply of 4v/9v HPV vaccines, you will let your daughter

A. Vaccinate with 2vHPV vaccines (domestic or imported) (Answer "36")

B. Not get vaccinated for the time being and wait until the 4v/9v HPV vaccines is available in the community (answer to "35")

C. Others________________ (please fill in) (answer to question "35")

35. How long will you be willing to wait in order for your daughter to receive a preferred type of HPV vaccine?

A. More than five years

B. Two to five years (including five years)

C. One to two years (including two years)

D. Six months to one year (including one year)

E. Six months or less

**Part D: Social Relationships**

The questions below describe many common situations in life. There are no right or wrong answers; the best answers are straightforward and spontaneous. Please read each question carefully and fill in the number that reflects your first response on the horizontal line following the question.

1 = never/almost never

2=rarely

3=Sometimes

4=Frequently

5=always/almost always

36. I am happy to help my friends and colleagues at work and in life. __________

37. I will share things about myself with my friends. __________

38. I will try to help others. __________

39. I can participate in volunteer activities to help those in need. __________

40. I will sympathies with those in need. __________

41. I will help those in need immediately. __________

42. I will do what I can to help others avoid getting into trouble. __________

43. I can feel strongly about what others are thinking. __________

44. I am willing to share what I have learned with others. __________

45, I try to comfort those who are sad. __________

46. I lend money or other things to people easily. __________

47. It is easy for me to think of those who feel upset. __________

48. I try to approach and look after those in need. __________

49. I easily share good opportunities that come my way with friends. __________

50. I will spend time with friends who feel lonely. __________

51. I notice my friends' discomfort immediately, even if this is not communicated directly to me. ________

**Questionnaire for the HPV Vaccination Promotion Project for Adolescent girl’s aged 15-18 years (Standard-of-care arm)**

ID No:

Adolescent girl’s name:

**Part A: Basic Information**

1. Your gender:

A. Male

B. Female

2. Your ethnicity:

A. Han

B. Others, please specify: __________

3. Your birth date __________ (e.g. "1990.01.01")

4. Your marital status is

A. Unmarried

B. Engaged or married

C. Separated or divorced

D. Widowed

E. Others, please specify: __________

5. The kinship between you and your child is

A. Father

B. Mother

C. Grandfather or grandfather

D. Grandmother or grandmother

E. Others, please specify: __________

6. Your highest education level is

A. Primary school or below

B. Junior high school

C. High school

D. Bachelor's degree or college

E. Postgraduate or above

7. Your current occupation is

A. Civil servant

B. Farmer

C. Ordinary worker (blue collar or labor-intensive work)

D. Company employee (white collar or office work)

E. Technician

F. Unemployed or retired

G. Others, please specify: __________

8. The annual income of your family is

A. 0-10,000 CNY/year (0-1,415 USD/Year)

B. 10000-30000 CNY /year (1,415-4,354 USD/Year)

C. 30,000-80000 CNY /year (4,354-11,611USD/Year)

D. 80000-150000 CNY /year (11,611-21,771USD/Year)

E. 150,000-300,000 CNY /year (21771-43541USD/Year)

F. 300000-1000000 CNY /year (43,541-145,137USD/Year)

G. More than 1,000,000 CNY /year (145,137 USD /Year or above)

9. Whether anyone in the same household has ever been infected with HPV?

A. Yes

B. No

C. No clear

10. Whether anyone in the same household has ever been infected with cancer cervical?

A. Yes

B. No

C. No clear

**Part B: Intention to receive vaccination**

11. The researchers have introduced you to the HPV vaccine today, are you willing to have your child vaccinated?

A. Yes

B. No (please skip to question 13)

12. If you are willing to have your child vaccinated against HPV, what are the reasons? [Multiple choice]

A. There are plans to get the HPV vaccine

B. It will protect my child from cervical cancer

C. Suggestion from a friend/family member

D. Recommendation from a medical professional

E. Other, please specify: _________

13. If you are reluctant to have your child vaccinated against HPV, what are the reasons? (If yes to question 11, then skip this question) [Multiple choice]

A. Don't know enough about cervical cancer and HPV vaccine

B. Unsure of the effectiveness of the HPV vaccine

C. Family members do not allow vaccination

D. Too much trouble

E. Worried about side effects

F. Price reason

G. Other, please specify: _________

**Part C: Vaccine-related information**

14. Have you ever heard about the HPV prior to this program?

A. Yes

B. No

15. Have you ever heard about HPV vaccines prior to this program?

A. Yes

B. No

16. Have ever heard the piloting projects to cover the HPV vaccination of 13-14 years old girls in Chengdu at the end of 2021?

C. Yes

D. No

17 Were you aware of the following vaccines marketed in this country prior to this program

|  | A. Yes | B. No |
| --- | --- | --- |
| 9v |  |  |
| 4v |  |  |
| 2v* |  |  |

*2v: both imported and domestic (same applies to later questions)

18. In general, I think HPV vaccine is important.

|  | A strongly disagree | B disagree | C agree | D strongly agree |
| --- | --- | --- | --- | --- |
| 9v |  |  |  |  |
| 4v |  |  |  |  |
| 2v* |  |  |  |  |

19. In general, I think the HPV vaccine is safe.

|  | A strongly disagree | B disagree | C agree | D strongly agree |
| --- | --- | --- | --- | --- |
| 9v |  |  |  |  |
| 4v |  |  |  |  |
| 2v* |  |  |  |  |

20. In general, I think the HPV vaccine is effective.

|  | A strongly disagree | B disagree | C agree | D strongly agree |
| --- | --- | --- | --- | --- |
| 9v |  |  |  |  |
| 4v |  |  |  |  |
| 2v* |  |  |  |  |

21. Has anyone around you been vaccinated against HPV?

A. Yes

B. No

22. Have you ever been hesitant about getting the HPV vaccination (except for allergies)?

A. Yes

B. No

23. Have you ever postponed the HPV vaccination (except for allergies)?

A. Yes

B. No

24. Have you ever refused to receive the HPV vaccination (except for allergies)?

A. Yes

B. No

25. Have you ever heard about negative information about HPV vaccination?

A. Yes

B. No

26. Do any of your friends or relatives object to HPV vaccination?

A. Yes

B. No

27. Have you or people around you had experienced adverse effects associated with HPV vaccination?

A. Yes

B. No

28. Is the price of the vaccine a barrier to HPV vaccination for you or others around you?

A. Yes

B. No

29. Do you think that, after HPV vaccination, your child still needs regular cervical cancer screening after they get married/become sexually active?

A. Yes

B. No

30. Which of the above three HPV vaccines would you preferably want your daughter to receive?

A. 2v-HPV vaccines (domestic or imported), because ________ (answer to "33")

B. Imported 4-HPV vaccines, because ________ (answer to question "31")

C. Imported 9-HPV vaccines, because ________ (answer to question "31")

D. No preferences, all of the above are fine (answer "33")

E. Not sure (answer "33")

31. If your community health center has no supply of 4v/9v HPV vaccines, you will let your daughter

A. Vaccinate with 2vHPV vaccines (domestic or imported) (Answer "33")

B. Not get vaccinated for the time being and wait until the 4-valent/9-valent vaccine is available in the community (answer to "32")

C. Others ________________ (please fill in) (answer "33")

32. How long will you be willing to wait in order for your daughter to receive a preferred type of HPV vaccine?

A. More than five years

B. Two to five years (including five years)

C. One to two years (including two years)

D. Six months to one year (including one year)

E. Six months or less

**Part D: Social Relationships**

The questions below describe many common situations in life. There are no right or wrong answers; the best answers are straightforward and spontaneous. Please read each question carefully and fill in the number that reflects your first response on the horizontal line following the question.

1 = never/almost never

2=rarely

3=Sometimes

4=Frequently

5=always/almost always

33. I am happy to help my friends or colleagues at work and in life. _________

34. I will share things about myself with my friends. _________

35. I will try to help others. _________

36. I can participate in volunteer activities to help those in need. _________

37. I will sympathies with those in need. _________

38. I will help those in need immediately. _________

39. I will do what I can to help others avoid getting into trouble. _________

40. I can feel strongly about what others are thinking.

41. I am willing to share what I have learned with others. _________

42, I try to comfort those who are sad. _________

43, I can easily lend money or other things to others. _________

44, It is easy for me to think about those who are feeling upset. _________

45, I try to approach and look after those in need. _________

46. I easily share good opportunities that come my way with friends. _________

47. I will spend time with friends who feel lonely. _________

48. I notice my friends' discomfort immediately, even if this is not communicated directly to me. _________
