# Supplementary material for: Factors affecting caregivers’ HPV vaccination decisions for adolescent girls: A secondary analysis of a Chinese RCT
Source: PLoS One. 2025 Jun 17;20(6):e0324260. doi: 10.1371/journal.pone.0324260 (PMC12173375; doi:10.1371/journal.pone.0324260)
Supplement: S1 Table — (DOCX) [file pone.0324260.s002.docx]

**S1Table. Collinearity diagnosis (N=321)**

| **Explanatory variables*** | **VIF** | **Tolerance** |
| --- | --- | --- |
| Age of the caregiver | 1.278 | 0.782 |
| Gender of the caregiver | 1.197 | 0.835 |
| Education | 1.406 | 0.711 |
| Have you ever heard of HPV | 2.890 | 0.346 |
| Have you ever heard of the HPV vaccine | 3.054 | 0.327 |
| Vaccine delay intention | 1.109 | 0.902 |
| Is price a barrier | 1.139 | 0.878 |
| Annual family income | 1.407 | 0.711 |
| Occupation | 1.200 | 0.833 |
| Pay-it-forward | 1.084 | 0.922 |
| Has HPV vaccination been delayed for some reason (other than allergy or illness) | 1.387 | 0.721 |
| Whether HPV vaccination was refused for some reason (other than allergy or illness) | 1.365 | 0.733 |
| Is there anyone around who has been vaccinated against HPV | 1.274 | 0.785 |
| Adverse reactions to HPV vaccination of friends or relatives | 1.128 | 0.886 |

*Explanatory variables selected for collinearity diagnosis were those variables with significant criteria<0.2 after the adjustment of prior confounder
