# Supplementary material for: Factors affecting caregivers’ HPV vaccination decisions for adolescent girls: A secondary analysis of a Chinese RCT
Source: PLoS One. 2025 Jun 17;20(6):e0324260. doi: 10.1371/journal.pone.0324260 (PMC12173375; doi:10.1371/journal.pone.0324260)
Supplement: S1 Fig — (DOCX) [file pone.0324260.s003.docx]

**S1 Fig. Caregivers’ willingness to vaccinate girls against HPV and girls’ actual vaccination rate**
