# Supplementary material for: Factors affecting caregivers’ HPV vaccination decisions for adolescent girls: A secondary analysis of a Chinese RCT
Source: PLoS One. 2025 Jun 17;20(6):e0324260. doi: 10.1371/journal.pone.0324260 (PMC12173375; doi:10.1371/journal.pone.0324260)
Supplement: Attachment — Submitted filename: Response to Reviewers.docx [file pone.0324260.s005.docx]

Feb 16^th^,2025

Dear Editors and Reviewers,

Thank you for your letter and for the Reviewers’ thoughtful and constructive comments regarding our manuscript entitled “Factors Influencing Caregivers’ Action to Vaccinate Adolescent Girls Against HPV in China” (PONE-D-24-51311).

We deeply appreciate the valuable feedback provided, which has been instrumental in revising and improving our manuscript. The comments have not only enhanced the quality of our paper but also provided significant guidance for our ongoing research.

We have carefully addressed each comment and made revisions that we hope will meet with your approval. The revised portions are marked in red in the manuscript for ease of review. Below, we provide a detailed summary of the main corrections made and our point-by-point responses to the Reviewers’ comments.

**Journal Requirements:**

We have thoroughly reviewed PLOS ONE’s style guidelines and ensured that our manuscript complies with all the formatting requirements.

As requested, we have amended the Role of Funder statement in the Funding section (line 396–397) to: “The funders had no role in study design, data collection and analysis, decision to publish, or preparation of the manuscript.” We have also updated the Funding Statement to: “The work reported in this publication is part of the research ‘Innovation Lab of Work Vaccine Delivery Research,’ supported by the Bill & Melinda Gates Foundation (INV-034554). The conclusions and opinions expressed in this work are those of the authors and shall not be attributed to the Foundation. Under the grant conditions of the Foundation, a Creative Commons Attribution 4.0 License has already been assigned to the Author Accepted Manuscript version that might arise from this submission.” We would appreciate your assistance in updating the online submission form accordingly.

In accordance with your request, the minimal de-identified data set necessary to replicate our study findings has been uploaded to a stable, public repository. The data can be accessed at [figshare] via the following link: [DOI:10.6084/m9.figshare.28424924]. We have also updated the Data Availability statement in the submission form accordingly.

**Reviewers’ comments:**

**Reviewer #1:** This study addresses a critical public health issue by investigating factors influencing HPV vaccination uptake among adolescent girls in China, a country facing significant cervical cancer burden. The findings provide valuable insights into caregiver decision-making and the impact of innovative interventions like the "pay-it-forward" model. However, methodological clarity, result interpretation, and presentation need improvement. I recommend a Major Revision.
Abstract
- Clarify the "pay-it-forward" intervention in a single concise sentence for accessibility to a broader audience.

RESPONSE：We greatly appreciate this valuable comment. In response, we have added the definition of “pay-it-forward” in the Methods section of the abstract: “Pay-it-forward is a novel model that motivates participants in adopting healthy behaviors and making community contributions. In this study, it offers an individual a free shot of HPV vaccine and then asks whether they would like to donate to support another person to get the same vaccination.” (line 26-29).

Introduction

- Clearly define the novelty of this study compared to existing research.

RESPONSE: We appreciate the Reviewer’s valuable advice. We have clarified the novelty of this study in the background section. The revised sentence is:“This study addresses this gap by focusing on the actual decision-making process of caregivers, thus bridging the divide between willingness and action. In addition, the study introduces a pay-it-forward intervention as a novel approach to influence vaccination behavior, which has not been extensively studied in the context of HPV vaccination.”(line 81-84).

- Provide a brief overview of similar interventions globally to strengthen the justification for the "pay-it-forward" approach.

RESPONSE: Thank you for your insightful suggestion. To strengthen the justification for the "pay-it-forward" approach, we have expanded the background section to include examples of similar interventions globally. The revised text reads “Previous studies have demonstrated that the pay-it-forward approach effectively enhances health behavior engagement, including increasing screening rates for gonorrhea, chlamydia, and hepatitis among men who have sex with men, as well as improving influenza vaccination rates among children and the elderly. ^[16-18]^” (line 87-90).
Methods:

- Elaborate on the randomization process and how bias was minimized during participant allocation.

RESPONSE: Thank you for your helpful comment. We have expanded on the randomization process in the Methods section. The revised text reads: “We obtained a de-identified contact list of adolescent girls aged 15–18 years from the residential committee through community health center staff. This list included all girls within this age range who have registered their names and contacts information at the community health center. We then performed simple random sampling to select eligible girls by generating a random number for each individual using Microsoft Excel. These individuals were sorted in ascending order based on their assigned random numbers. Recruitment proceeded by contacting individuals at the top of the list until the desired sample size was reached at each study site.” (line 112-119).

Additionally, we have elaborated on how bias was minimized during participant allocation. The revised text now states： “Upon arrival at the study site, participants were randomly assigned to either the standard-of-care arm or the pay-it-forward arm using sealed opaque envelopes to ensure allocation concealment. Each envelope contained a random numerical ID corresponding to the assigned arm (standard-of-care or pay-it-forward). The allocated intervention was concealed from both study participants and research assistants onsite prior to assignment until the envelope was opened, thereby maintaining blinding throughout the assignment process.” (line 120-126).

- Clarify whether participants were blinded to the intervention groups to reduce bias.

RESPONSE: Thank you for your insightful comment. In the revised manuscript, we have clarified that participants were blinded to the intervention groups in our study. The revised text states: “Upon arrival at the study site, participants were randomly assigned to either the standard-of-care arm or the pay-it-forward arm using sealed opaque envelopes to ensure allocation concealment. Each envelope contained a random numerical ID corresponding to the assigned arm (standard-of-care or pay-it-forward). The allocated intervention was concealed from both study participants and research assistants onsite prior to assignment until the envelope was opened, thereby maintaining blinding throughout the assignment process.” (line 120-126).

- Provide more detail on the operationalization of "intention to delay vaccination"—include thresholds or criteria.

RESPONSE: We appreciate the reviewer’s insightful comment. In the context of China’s limited supply and difficulty in securing appointments for 4v- and 9v- vaccines, we operationalized the “intention to delay vaccination” by including a specific question in the questionnaire. Caregivers were asked whether they planned to wait for the 4v- or 9v-HPV vaccines despite the availability of the domestically produced 2v-HPV vaccine. Caregivers who indicated their intent to wait for the 4v- or 9v- HPV vaccines were categorized as intending to delay vaccination.

This operationalization is now discussed in detail in the "Independent variables" section. Additionally, to provide further context, we have added the following statement: “In the context of China’s limited supply and difficulty in securing appointments for 4v- and 9v- HPV vaccines.” (line 189-190) to elaborate further.

Results:

- Expand the discussion of multivariate regression results to link findings back to public health implications.

RESPONSE: Thank you for your helpful suggestion. In the revised manuscript, we have expanded the discussion of the multivariate regression results and explicitly linked the findings to public health implications. Specifically, we have provided a more detailed interpretation of how factors such as caregiver occupation, knowledge of the HPV vaccine, and the intention to delay vaccination were associated with HPV vaccine uptake. These findings highlight critical areas where targeted health interventions can address both individual and systemic barriers to HPV vaccination.

Additionally, we have emphasized the relevance of our findings for regions with limited government-funded vaccination programs, such as parts of Africa and South Asia, where access to HPV vaccines is restricted. Our study underscores the importance of strategies such as financial subsidies and tailored health education, which could help improve HPV vaccination uptake in these regions and ultimately reduce the burden of cervical cancer. The revised text states: “HPV vaccination rates remain low in many countries, particularly in low-income regions where vaccine access is limited and healthcare systems may lack the infrastructure to promote the vaccine effectively^[39]^. The significant role of economic barriers and lack of knowledge about the vaccine mirrors challenges observed in other countries, such as India and several sub-Saharan African nations^[40, 41]^. Although this study was conducted in China, the findings are highly relevant to other regions facing similar barriers to HPV vaccination uptake. Many low-and middle-income countries, particularly those with limited government-sponsored vaccination programs, could benefit from strategies that combine financial support with community-driven health education initiatives. These approaches address both financial and informational barriers, which are prevalent globally.” (line 335-345).

- Clearly indicate whether variables included in the final model were pre-specified or selected post hoc based on significance.

RESPONSE：Thank you for your valuable comment. The selection of variables for the final multivariable model was conducted post hoc and based on statistical significance after adjusting for caregivers' age and gender, as outlined in the statistical analysis section. Specifically, variables with a *P*-value less than 0.2 after adjustment were included in the model. This threshold was chosen to ensure that potentially important predictors were not excluded prematurely, while maintaining a balance between model complexity and interpretability. We have also emphasized this selection criterion in the results section, where it is stated: “Variables with a *P*-value less than 0.2 after adjusting for age and gender of caregivers were included in the multivariable model.” (line 246-247).

Discussion:

- Deepen the analysis of cultural stigma and its implications for vaccination programs.

RESPONSE: We appreciate the Reviewer’s comment and have expanded the analysis of cultural stigma in HPV vaccination within the discussion section. The revised text reads: “In addition to misinformation and vaccine availability, cultural stigma significantly impacts HPV vaccine uptake. In many societies, the HPV vaccine is often associated with sexual activity, leading to widespread misconceptions about its purpose and necessity. This stigma can deter caregivers from vaccinating adolescents due to concerns about societal judgment or misinterpretation of their intentions. To address this, public health campaigns should emphasize the vaccine’s role in preventing cervical cancer while incorporating culturally sensitive messaging while incorporating culturally sensitive messaging. These efforts can help to reduce stigma, promote acceptance, and improve vaccination uptake.” (line 325–334).

- Discuss global implications of the findings and their relevance beyond Iran.

RESPONSE: We appreciate the reviewer’s insightful comment. While this study was conducted in China, we have taken the suggestion into account and expanded the discussion to address the broader global implications of our findings. The following text has been added: “Although this study was conducted in China, the findings are highly relevant to other regions facing similar barriers to HPV vaccination uptake. Many low-and middle-income countries, particularly those with limited government-sponsored vaccination programs, could benefit from strategies that combine financial support with community-driven health education initiatives. These approaches address both financial and informational barriers, which are prevalent globally. Future research could explore how the“pay-it-forward” model might be adapted to improve HPV vaccination rates in diverse settings, tailoring the intervention to align with local context, cultural nuances, and available resources.” (line 339–348).

- Critically evaluate the study’s limitations, particularly its reliance on a small, urban sample and absence of quantitative data.

RESPONSE：We appreciate the reviewer’s valuable suggestion. In response, we have revised the limitations section to provide a more critical evaluation of the study’s sample and data limitations. The original sentence has been updated from: “As a study embedded within a randomized controlled trial, the sample size was limited and not specifically powered to assess all variables influencing vaccination behavior” to “As a study embedded within a randomized controlled trial, the sample size was relatively small and limited to an urban sample, which may restrict the generalizability of the findings to rural areas or other regions with different socio-cultural contexts.” (line 350-353). Our study is indeed a quantitative analysis. However, we understand that the reviewer’s concern may stem from the limited range of variables considered in the study. While our current model focused on the most significant factors (e.g., caregiver age, gender, and vaccine preferences), we acknowledge that the study could benefit from a more extensive set of quantitative variables, such as detailed socio-economic status, health literacy, and psychological factors, which could provide a more nuanced understanding of vaccination behavior.
- Suggest future research directions, such as mixed-method studies or larger cross-sectional analyses.

RESPONSE：We appreciate the reviewer’s valuable suggestion. We have revised the conclusion section to address your suggestion. The following text has been added: “Future research should aim to address these limitations by conducting larger, more diverse cross-sectional studies to improve the representativeness of findings. Mixed-methods studies combining quantitative and qualitative approaches could also provide deeper insights into the cultural, socioeconomic, and psychological barriers to HPV vaccination.” (line 377–381).

Conclusion:

- Expand on the limitations of the study, particularly concerning sample size and potential biases due to the socioeconomic distribution of participants.

RESPONSE: Thank you for your valuable feedback. We have revised the conclusion section to address your suggestion. The following text has been added: “However, there are several limitations in this study. The relatively small and urban-focused sample size may restrict the generalizability of our findings to rural or socioeconomically diverse populations. Additionally, potential biases may arise from the socioeconomic distribution of participants, which could influence the observed associations.” (line 373–377).

- Discuss how the findings align or diverge from global HPV vaccination trends.

RESPONSE: Thank you for your insightful comment. In response to your suggestion, we have expanded our discussion to better contextualize our findings within global HPV vaccination trends. Specifically, we have highlighted that “HPV vaccination rates remain low in many countries, particularly in low-income regions where vaccine access is limited and healthcare systems may lack the infrastructure to promote the vaccine effectively. The significant role of economic barriers and lack of knowledge about the vaccine mirrors challenges observed in other countries, such as India and several sub-Saharan African nations.” (line 339-333). Additionally, we have included a discussion on how the findings diverge from global HPV vaccination trends, noting that “Caregivers who intended to delay HPV vaccination were less likely to vaccinate their adolescent daughters, suggesting that the intention to delay vaccination may be a particular and specific barrier in China.” (line 306-308).
- Provide actionable recommendations for public health policymakers based on the findings.

RESPONSE: Thank you for your valuable feedback. We have revised the conclusion to make the recommendations more specific and actionable for public health policymakers. The updated version emphasizes targeted health education for underserved communities, financial subsidies for low-income families, and community involvement through local health workers. These adjustments address your concerns and enhance the clarity of the policy recommendations. The original sentence: “To enhance vaccination rates, it is crucial to implement tailored health education initiatives that address specific knowledge gaps and misconceptions. Additionally, providing financial subsidies can alleviate economic barriers, while fostering community involvement will strengthen trust and engagement among caregivers. Future efforts to improve HPV vaccination must prioritize these strategies, as they have the potential to significantly increase vaccination rates and contribute to cervical cancer prevention in both China and similar public health contexts” has been revised to “These findings highlight the importance of tailored health education initiatives that address specific knowledge gaps and misconceptions, particularly for caregivers in underserved communities. Providing financial subsidies remains a critical strategy to alleviate economic barriers, especially for low-income families. Furthermore, fostering community involvement through local health workers can strengthen trust and engagement among caregivers. By leveraging these strategies, future public health efforts can further refine interventions to significantly enhance HPV vaccination rates and contribute to cervical cancer prevention, both in China and in similar public health contexts globally.” (line 364-372).

**Reviewer #2:** This study, carried out in China with a relatively large N: Factor Influencing Caregivers' Action to Vaccinate Adolescent Girls Against HPV in China, is fairly well described. The method is replicable, and the process and outcome of the study are well understood. Nevertheless, several parts of the article are unclear and lack understanding for the reader.
my comments to make the article easier to read and understand:

Title: the study design should be added to the title.

RESPONSE: We appreciate the reviewer’s suggestion and have implemented the recommended change. In response, we have revised the title to include the study design for greater clarity and to better reflect the nature of the research. The original title: “Factors Influencing Caregivers” Action to Vaccinate Adolescent Girls Against HPV” has been revised to: “Factors Influencing Caregivers’ Action to Vaccinate Adolescent Girls Against HPV: Secondary Analysis of a Randomized Controlled Trial in China”

Abstract: in the method section, explain how the data was collected.

ESPONSE: We appreciate the Reviewer’s valuable suggestion and have revised the method section of the abstract for greater clarity regarding data collection. We stated in the abstract methods section that sociodemographic data were collected through questionnaires and supplemented with the method of collecting information on the receipt of the first dose of the HPV vaccine. The original sentence: “The endpoint was the receipt of the first dose HPV vaccine following an intervention or no intervention (pay-it-forward vs. standard-of-care)” has been revised to: “The endpoint was the receipt of the first dose HPV vaccine following an intervention or no intervention (pay-it-forward vs. standard-of-care), which was obtained from an electronic vaccination registry system.” (line 36-37).

Line 75 - 82: this section should go into the method section, to finish the background with the study objective.

RESPONSE: We appreciate the reviewer’s suggestion and have revised the manuscript accordingly. The section previously located in line 75–82, which discussed the study design and background of the trial, has been relocated to the Methods section to ensure better organization and flow. In the revised manuscript, we now conclude the Background section with a succinct statement of the study’s objective:“This study aims to investigate factors influencing caregivers’ action to vaccinate their adolescent girls, based on a pay-it-forward intervention program designed to improve HPV vaccine uptake among adolescent girls in China.” (line 91-93).

Methods:

Even if the entire protocol has been published elsewhere (ref 16), certain information should be given in this article. In particular, the calculation of the sample size and the expected N of the study to see if the power of the study has been respected.

RESPONSE: We appreciate the reviewer’s comment and have included the requested information regarding the sample size calculation in the revised manuscript. The following text has been added: “The sample size was estimated based on a pilot study conducted in Chengdu from January to February 2022. In this pilot study, vaccine uptake was observed to be 98% in the pay-it-forward arm and 82% in the standard-of-care arm, with a 16% difference. However, due to the low HPV vaccination rate (<10%) in the target population, we conservatively estimate 20% uptake in the standard-of-care arm and 36% in the pay-it-forward arm for the trial. To detect this difference with 80% power, 120 participants are required per arm. Considering a 10% non-response or dropout rate, 133 participants per arm are needed. To allow for secondary and subgroup analyses, the desired sample size was further increased by 20%, resulting in a total of 320 participants.” (line 138–149).

Line 119-122: this part should go in an ethics section at the end of the method.

RESPONSE: Thank you for the reviewer’s suggestion. We have moved the ethical approval information previously located in line 215–219 to a dedicated “2.5 Ethical approval” section at the end of Methods. This adjustment ensures that ethical details are presented separately and clearly, improving the structure and readability of the manuscript.

Table 2: table 2 in its current format is very difficult to read, and it's hard to see which variable is linked to which OR. Statistically significant ORs should be highlighted more prominently. Since we have the 95% CIs, the statistical p's are not useful, which would save space and visibility in the table.

RESPONSE: Thank you for your helpful suggestion. We have revised Table 2 to improve readability and clarity. Specifically: 1. Improved Formatting: Variables have been bolded to clearly indicate their association with the corresponding odds ratios (ORs), enhancing the table's organization and readability. 2. Statistical Significance Highlighted: Statistically significant ORs are now bolded to make them more prominent, highlighting key findings at a glance. While we acknowledge that the 95% confidence intervals (CIs) provide important information about the precision of the estimates, we believe that *P*-values remain valuable in interpreting the statistical significance of the results. *P*-values are widely used to assess the strength of evidence against the null hypothesis and complement the ORs and CIs in conveying the statistical significance. To maintain transparency and clarity in our findings, we have chosen to retain the P-values in the table, as they serve as an essential part of the statistical interpretation alongside the ORs and CIs. We believe these changes improve the table's visibility and utility for readers.

The discussion is complete and understandable, but I think it should be structured into sub-chapters, with a clearly defined and developed limit of the study.

RESPONSE: Thank you for your suggestion. We have carefully considered structuring the discussion into sub-chapters. However, we believe that the discussion flows more cohesively as a single section, as the content is closely interconnected and addresses the findings in a unified narrative. To address your concern, we have created a separate subsection specifically for the study limitations, allowing us to clearly define and develop them without interrupting the overall flow of the discussion. It reads as “Our study has several limitations. As a study embedded within a randomized controlled trial, the sample size was relatively small and limited to an urban sample, which may restrict the generalizability of the findings to rural areas or other regions with different socio-cultural contexts. Additionally, the intervention at one site coincided with the implementation of new COVID-19 guidelines, potentially affecting participants’ willingness to visit clinics and thereby influencing vaccination rates. Frequent stockouts of the 4v- and 9v- HPV vaccines may have also led to underestimation of vaccine uptake due to this structural barrier. Nonetheless, alternative HPV vaccine options were available across all sites, ensuring access to vaccination services for participants willing to consider other formulations.” (line 350-359). We believe this structure enhances clarity and readability while ensuring the limitations are appropriately emphasized.

We also have successfully uploaded all relevant figure files to the Preflight Analysis and Conversion Engine (PACE) digital diagnostic tool, as requested.

We have made every effort to improve the manuscript and have implemented the suggested changes. These revisions do not alter the content or framework of the paper but rather enhance its clarity and readability. We sincerely appreciate the time and effort the Editors and Reviewers have dedicated to providing insightful comments and suggestions. We hope that these corrections meet your expectations and receive your approval.

Once again, thank you for your invaluable feedback and guidance.

Sincerely,

Dr. Jing Li

Department of Epidemiology and Statistics, West China School of Public Health and West China Fourth Hospital, Sichuan University, Chengdu, China, 610041

Email: [lijingwcph@scu.edu.cn](mailto:lijingwcph@scu.edu.cn)
