# Supplementary material for: Factors affecting caregivers’ HPV vaccination decisions for adolescent girls: A secondary analysis of a Chinese RCT
Source: PLoS One. 2025 Jun 17;20(6):e0324260. doi: 10.1371/journal.pone.0324260 (PMC12173375; doi:10.1371/journal.pone.0324260)
Supplement: Attachment — Submitted filename: Response to Reviewers-second.docx [file pone.0324260.s006.docx]

Mar 3^th^,2025

Dear Dr. Emily Chenette,

I am writing to resubmit our manuscript originally titled “Factors Influencing Caregivers’ Action to Vaccinate Adolescent Girls Against HPV: Secondary Analysis of a Randomized Controlled Trial in China”, now revised and retitled “Factors Affecting Caregivers’ HPV Vaccination Decisions for Adolescent Girls: A Secondary Analysis of a Chinese RCT”, for consideration for publication in *PLOS One*. The manuscript ID for the initial submission was PONE-D-24-51311R1.

In accordance with the editor’s decision letter, we have thoroughly addressed the issues raised by the reviewers and believe that our manuscript is now significantly improved. We have included a point-by-point response to the reviewers’ comments as detailed below. All changes are highlighted in red in the manuscript for your convenience.

Reviewers’ comments:

Reviewer #4: This manuscript focuses on investigating factors influencing caregivers' decisions to vaccinate adolescent girls against HPV in China through a secondary analysis of a randomized controlled trial. The study has addressed some important public health issues and has been revised based on reviewers' comments, but there is still room for improvement in multiple aspects. Here are detailed revision suggestions:

Title and Abstract

Title: The addition of "Secondary Analysis of a Randomized Controlled Trial in China" in the title clarifies the study design, which is a good improvement. However, it could be made more concise. Consider rephrasing to something like "Factors Affecting Caregivers' HPV Vaccination Decisions for Adolescent Girls: A Chinese RCT Secondary Analysis" to enhance readability.

RESPONSE: Thank you for your insightful feedback on the title. We agree that a more concise title would improve readability. In response, we have revised the original title from “Factors Influencing Caregivers’ Action to Vaccinate Adolescent Girls Against HPV: Secondary Analysis of a Randomized Controlled Trial in China” to “Factors Affecting Caregivers’ HPV Vaccination Decisions for Adolescent Girls: A Secondary Analysis of a Chinese RCT.”

Abstract: While the definition of the "pay - it - forward" intervention and data collection method have been added, it could be further improved. For example, briefly mention the key results more quantitatively. Instead of just stating the factors associated with vaccination uptake, provide approximate percentages or odds ratios in a more summary - oriented way to give readers a quicker understanding of the magnitude of the effects.

RESPONSE: We appreciate the reviewer’s valuable suggestion and have made the recommended adjustments to the abstract for greater clarity. Specifically, we have incorporated a more concise summary of the key results with quantitative details. The revised sentence is as follows:“Daughters of caregivers who were previously unaware of the HPV vaccine were three times more likely to be vaccinated (*OR*=3.01, 95% *CI*: 1.27-7.14). Caregivers who did not intend to delay vaccination had daughters with eight times higher vaccination uptake (*OR*=8.26, 95% *CI*: 4.36-15.67). Participation in the ‘pay-it-forward’ intervention increased vaccination rates by more than twofold (*OR*=2.22, 95% *CI*: 1.19-4.15). Daughters of unemployed or retired caregivers had nearly four times higher vaccination rates compared to those whose caregivers were employed (*OR*=3.97, 95% *CI*: 1.81-8.72). Prior refusal of the vaccine by caregivers was associated with an 80% reduction in vaccination uptake among daughters (*OR*=0.23, 95% *CI*: 0.06-0.81).” (lines 40-49)

Introduction

Literature Review: Although the background provides an overview of the HPV vaccination situation in China and the importance of the study, it could be more comprehensive. Cite more recent and diverse literature, especially studies that have explored the gap between willingness and action in HPV vaccination in different cultural or geographical contexts. This would strengthen the justification for the current study.

RESPONSE: Thank you for your insightful feedback on the literature review section. We appreciate your suggestion to provide a more comprehensive overview and to include a broader range of recent, diverse literature, particularly studies that explore the gap between willingness and action in HPV vaccination across different cultural and geographical contexts. In response, we have revised the literature review to incorporate additional recent studies, expanding the scope to include research from various regions and populations that address this gap. This inclusion helps strengthen the justification for our study by contextualizing the findings in a broader, global framework.

The following text has been added to enhance the discussion:“Recent studies from regions such as India, Southeast Asia, and Europe have highlighted similar gaps between vaccine willingness and actual uptake^[16-18]^. These findings underscore the importance of understanding not only the factors that influence willingness to vaccinate, but also the broader socio-cultural, economic, and healthcare system factors that play a critical role in shaping actual vaccine uptake.” (lines 79-84)

Research Gap: The statement of the research gap is clear, but it could be more specific. Explain precisely how the "pay - it - forward" intervention fills the gap in the existing literature, not just in terms of influencing vaccination behavior but also in comparison to other similar interventions that may have been tried in the HPV vaccination field.

RESPONSE: Thank you for your constructive feedback. In response to your suggestion, we have revised the manuscript to more explicitly describe how the "pay-it-forward" intervention addresses gaps in the existing literature. The updated explanation clarifies the unique contribution of this intervention compared to other strategies in the HPV vaccination field.

The original sentence was revised from:“In the 'pay-it-forward' model, an individual receives a gift (e.g., a subsidized HPV vaccine) and is then asked whether they would like to donate to support another person. Previous studies have demonstrated that the pay-it-forward approach effectively enhances health behavior engagement, including increasing screening rates for gonorrhea, chlamydia, and hepatitis among men who have sex with men, as well as improving influenza vaccination rates among children and the elderly^[16-18]^.”to:“While various interventions, such as health education and financial incentives, have been explored to increase HPV vaccination uptake, they have often faced limitations in overcoming socio-cultural barriers and promoting sustained behavior change^[19, 20]^. In contrast, the “pay-it-forward” model not only provides a tangible benefit (e.g., a subsidized HPV vaccine) but also leverages social reciprocity by encouraging individuals to donate or support others. This unique mechanism taps into intrinsic social motivations, which has shown effectiveness in improving engagement in other health behaviors, such as increasing STI screening rates among men who have sex with men and flu vaccination rates among children and elderly populations ^[21-23]^. The novelty of this intervention lies in its potential to bridge the gap between willingness and actual vaccine uptake by aligning social norms and altruistic behavior with public health goals.” (lines 91-103)

Methods

Study Design and Participants: The randomization process and blinding description have been improved. However, it would be beneficial to provide more details about the characteristics of the four community health service centers in Chengdu. For example, mention their location (urban/rural distribution, socioeconomic characteristics of the surrounding areas), as this could potentially influence the results and the generalizability of the findings.

RESPONSE: We sincerely thank the reviewer for this constructive suggestion. To provide greater context on the characteristics of the four community health service centers in Chengdu and their potential influence on the results and generalizability of our findings, we have made the following revisions to the "Study Design and Participants" section: “The four sites were selected to reflect varying socio-economic contexts based on the relative level of individual disposable income in 2021. This approach ensures a broad representation of different socio-economic environments within Chengdu. The selected sites are as follows: Site A: Located in the most developed urban area, this site benefits from higher healthcare access and represents a higher socio-economic status with better public health infrastructure. Site B: Situated in a higher middle-income suburban area, this site experiences relatively good healthcare access but is influenced by suburban socio-economic factors. Site C: This site represents a lower middle-income suburban area with more limited healthcare resources and socio-economic challenges. Site D: The least developed area in terms of infrastructure, healthcare access, and socio-economic conditions. This site faces the greatest challenges related to economic factors and healthcare delivery.” (lines 122-133)

Sample Size Calculation: The addition of sample size calculation details is a great improvement. To further enhance transparency, explain why a 10% non - response or dropout rate was assumed. Provide some references or prior data to support this assumption.

RESPONSE: We sincerely thank the reviewer for this helpful suggestion. We have provided additional details regarding the basis for the 10% non-response or dropout rate assumption to enhance transparency. The 10% dropout rate was primarily informed by the findings from the pre-test conducted in our pilot study in Chengdu. In this pilot phase, we observed a certain level of participant attrition, with approximately 10% of participants not completing the study. Given the context of our study, where participants may experience logistical or personal challenges in completing the trial, this pilot data provided a conservative estimate for the dropout rate. While we did not find direct literature references for a 10% dropout rate specific to similar studies, the pilot data provided a reasonable estimate to ensure adequate statistical power.

We have now incorporated this additional explanation in the revised manuscript. The original sentence has been updated from: “Considering a 10% non-response or dropout rate, 133 participants per arm are needed.” to: “Considering a 10% non-response or dropout rate based on results from the pilot study, 133 participants per arm are needed.” (line 172).

Variable Measurements: For the "intention to delay vaccination" variable, while the operationalization is now clear, consider validating this measure. You could mention if there were any pilot studies or pre - testing of this question to ensure that it accurately captures the concept. Also, for other variables, such as "vaccine hesitancy," describe in more detail how the responses were coded and categorized.

RESPONSE: Thank you very much for your insightful comments regarding the operationalization and validation of the "intention to delay vaccination" and "vaccine hesitancy" variables. We appreciate your suggestions and have revised the manuscript to address them in more detail.

Intention to Delay Vaccination:

To ensure that the "intention to delay vaccination" variable accurately captures the intended concept, we conducted a pilot study prior to the main trial. In this pilot study, we pre-tested the survey instrument with a small, diverse sample of participants from the target population to assess the clarity, comprehensibility, and relevance of the questions. Based on the feedback received, adjustments were made to enhance the clarity and specificity of the wording. We have added the following text to the manuscript to clarify this process:“To ensure that this measure accurately captures the concept of intention to delay vaccination, we conducted a pilot study in which the wording of this question was pre-tested for clarity and understanding with a small group of caregivers from the study’s target population.” (lines 216-219).

Vaccine Hesitancy:

Vaccine hesitancy was assessed by retrospectively examining whether participants had ever experienced hesitation, postponement, or refusal of HPV vaccination. Specifically, participants were asked the following questions:“Have you ever postponed the HPV vaccination (except for allergies)?”“Have you ever refused to receive the HPV vaccination (except for allergies)?”“Have you ever heard negative information about HPV vaccination?” These questions were designed to capture past instances of vaccine-related hesitation and decision-making rather than to determine an overall hesitancy score. Each response was recorded separately to provide insights into different aspects of past vaccine-related behaviors. This approach ensures a clear and detailed understanding of participants' prior experiences with HPV vaccination. We have updated the manuscript to include this clarification: “Vaccine hesitancy was assessed by asking participants to indicate their level of agreement with three statements regarding past behavior and attitudes: “Have you ever been hesitant about getting the HPV vaccination (except for allergies)?”; “Have you ever postponed the HPV vaccination (except for allergies)?”; and “Have you ever refused to receive the HPV vaccination (except for allergies)?” Each response recorded separately to reflect different aspects of past vaccine-related decision-making.” (lines 228-234).

Statistical Analysis: Clearly state the assumptions of the logistic regression models. For example, discuss the assumption of independence of observations and how it was met in the context of this study. Also, explain why a backward stepwise strategy was chosen over other variable selection methods, and discuss its potential limitations.

RESPONSE: Thank you for your insightful comment regarding the assumptions of the logistic regression models and the rationale behind the selection of the backward stepwise method. In response to your suggestion, we have revised the manuscript to provide a more detailed explanation of the assumptions underlying the logistic regression models, as well as a clearer justification for the choice of the backward stepwise strategy.

Assumptions of Logistic Regression Models:

We appreciate your suggestion to elaborate on the assumptions made when applying logistic regression models. The following text has been added to the manuscript to clearly outline the assumptions:“The logistic regression models in this study assumed the independence of observations, linearity of the logit, and the absence of multicollinearity. To ensure the independence of observations, participants were randomized at the individual level, and no repeated measures were taken from the same individual.” (line 249-253).

Rationale for Backward Stepwise Strategy and Potential Limitations:

The backward stepwise method was chosen for its systematic approach to model selection, in which non-significant variables are iteratively removed to achieve a more parsimonious and interpretable model. This strategy helps identify the most important predictors and eliminates irrelevant variables, thereby improving model simplicity and interpretability. We have added the following text to justify this choice:“This method was chosen for its systematic elimination of non-significant variables and ability to simplify the model. However, it carries risks of overfitting, exclusion of important interactions, and dependence on the initial variable set.” (lines 246-249).

Results

Presentation of Results: The results section is generally well - presented. However, in Table 1, provide more information about the missing data. Report the number of missing values for each variable, and if possible, discuss how missing data were handled. In Table 2, consider adding a column to show the direction of the association (e.g., positive or negative) for each variable, especially for readers who may quickly scan the table.

RESPONSE: Thank you for your valuable feedback on the presentation of the results. In response to your suggestions, we have made the following revisions to improve transparency and readability:

1. Missing Data in Table 1: We appreciate your suggestion to provide more detailed information about missing data in Table 1. In the revised manuscript, we have included the number of missing values for the "age" variable. Specifically, there were 19 missing values for this variable, which accounts for a very small proportion of the total sample. We did not observe missing data for any other variables in the dataset, and therefore, no imputation or special handling was required. We have updated the table’s note to clearly indicate this information:“There were 19 missing values for the variable ‘age’.” (line 278).
2. Direction of Association in Table 2: In response to your suggestion to include a column indicating the direction of the association in Table 2, we have added a new column titled "Direction of Association." This column shows whether the association between each variable and the outcome is positive or negative, based on the odds ratio (OR). Specifically, we classified the association as:Positive if the OR > 1, indicating a positive association with the outcome; Negative if the OR < 1, indicating a negative association with the outcome.

Interpretation of Results: Expand on the interpretation of the results in the text. For example, when discussing the significant factors, compare the magnitudes of the odds ratios in more detail. Explain why the effect of the "pay - it - forward" intervention on vaccination uptake is of a certain magnitude, and how it compares to other factors in practical terms.

RESPONSE:

Thank you for your insightful feedback on the interpretation of the results. In response to your suggestion to provide a more detailed interpretation of the significant factors and compare the magnitudes of the odds ratios (*OR)*, we have made the following revisions to enhance the discussion:

1. Comparison of Odds Ratios (*OR)*: We have expanded on the interpretation of the odds ratios, particularly in terms of comparing the relative magnitudes of the significant factors influencing vaccination uptake. In the revised manuscript, we offer a detailed comparison of the *OR* for the “pay-it-forward” intervention (*OR* = 2.22) and other significant factors such as intention to delay vaccination (*OR* = 8.26). This comparison highlights the relative importance of these factors in predicting vaccination uptake. Specifically, we emphasize that while the “pay-it-forward” intervention has a notable positive effect on vaccine uptake (*OR* = 2.22), its effect is not as strong as the intention to delay vaccination (*OR* = 8.26), which emerges as a key barrier to vaccination. This suggests that the “pay-it-forward” intervention plays a critical role in motivating individuals to vaccinate, but addressing the underlying barriers related to the intention to delay vaccination will be crucial for achieving higher vaccine uptake. We also explain that the pay-it-forward intervention's effect stems from its use of social reciprocity, which encourages individuals to not only benefit from the intervention but also feel compelled to "pay it forward" and support others. This approach leverages community trust and social norms to create a sense of responsibility, which helps overcome vaccine hesitancy and apathy.
2. Interpretation of Other Factors: We have added more detail on the impact of other factors, such as caregivers' employment status and awareness of the HPV vaccine. The findings suggest that unemployed or retired caregivers were more likely to vaccinate their daughters, which may indicate a higher level of trust in the healthcare system and the importance of informational campaigns. This insight provides valuable implications for targeting specific groups in future interventions.
3. Vaccine Hesitancy: We have included a new discussion on the caregivers who had previously refused the HPV vaccine (OR = 0.23). This subgroup was significantly less likely to change their stance and vaccinate their daughters, indicating that vaccine hesitancy remains a significant challenge. The low odds ratio for this group suggests that once caregivers refuse the vaccine, it becomes much more difficult to persuade them to reconsider, emphasizing the need for more intensive, targeted efforts to build trust in vaccine safety and efficacy.

These changes were made in lines 324– 396 of the revised manuscript.

Discussion

Comparison with Other Studies: The discussion of cultural stigma has been expanded, which is good. However, when comparing the study findings with other regions, provide more in - depth analysis. For example, compare the prevalence of cultural stigma - related barriers in China with those in other Asian countries or globally. Analyze how cultural differences may lead to different levels of stigma and its impact on vaccination rates.

RESPONSE: Thank you for your insightful suggestion to enhance the comparison of cultural stigma across regions. In response to your recommendation, we have expanded the discussion to provide a more comprehensive analysis of cultural stigma and its impact on HPV vaccine uptake. The original sentence has been revised as follows:

Original:“In addition to misinformation and vaccine availability, cultural stigma significantly impacts HPV vaccine uptake^[38]^. In many societies, the HPV vaccine is often associated with sexual activity, leading to widespread misconceptions about its purpose and necessity.”

Revised:“In addition to misinformation and vaccine availability, cultural stigma significantly impacts HPV vaccine uptake ^[43, 44]^. In China, cultural concerns about adolescent sexuality and the HPV vaccine’s association with sexual behavior contribute to vaccine hesitancy. Similar stigma is seen in other Asian countries, such as Hong Kong and South Korea, though its intensity varies. In India, seeking the HPV vaccine can be stigmatized due to concerns over family honor and social prestige, further fueling vaccine hesitancy ^[44]^.” (lines 385- 390)

Global Implications: The global implications section could be more focused. Instead of a broad statement about low - and middle - income countries, identify specific regions or countries where the "pay - it - forward" model could be most applicable. Provide some preliminary ideas on how the model might need to be adapted for different cultural, economic, and healthcare system contexts.

RESPONSE: Thank you for your thoughtful feedback regarding the global implications section. We agree that the section could benefit from a more focused discussion and appreciate your suggestion to identify specific regions or countries where the "pay-it-forward" model could be most applicable. In response, we have revised the section to pinpoint regions with prevalent financial and informational barriers to vaccination and to provide some preliminary ideas on how the model might be adapted to diverse cultural, economic, and healthcare contexts.

Original:“Many low- and middle-income countries, particularly those with limited government-sponsored vaccination programs, could benefit from strategies that combine financial support with community-driven health education initiatives. These approaches address both financial and informational barriers, which are prevalent globally.”

Revised: “Countries in Sub-Saharan Africa, Southeast Asia, and rural areas of Latin America, where financial and informational barriers to vaccination are prevalent, could benefit from strategies that combine financial support with community-driven health education initiatives. For example, in Southeast Asia, where there are often cultural stigmas related to adolescent sexuality, the ‘pay-it-forward’ model could be adapted by involving local leaders, such as religious figures or community health workers, to help reshape societal perceptions around the HPV vaccine.” (line 404- 411)

Limitations: The limitations section has been improved, but it could be more comprehensive. Discuss the potential impact of the COVID - 19 guidelines on the intervention in more detail. For example, how did the new guidelines specifically affect participants' willingness to visit clinics? Were there any differences in the impact across different groups of participants? Also, consider adding a limitation related to the self - reported nature of the data, as there may be recall bias or social desirability bias in caregivers' responses.

RESPONSE: Thank you for your valuable suggestions on expanding and clarifying the limitations section. We have revised the manuscript to address the potential impact of the COVID-19 guidelines on the intervention in more detail, as well as to incorporate a limitation related to the self-reported nature of the data.

Impact of COVID-19 Guidelines:

We have further elaborated on how the COVID-19 pandemic and related guidelines may have impacted participants' willingness to visit clinics. Specifically, the revised text now reads:

Original:“Additionally, the intervention at one site coincided with the implementation of new COVID-19 guidelines, potentially affecting participants’ willingness to visit clinics and thereby influencing vaccination rates.”

Revised: “Additionally, the intervention at Site D coincided with the COVID-19 pandemic, which may have reduced participants’ willingness to visit clinics due to restricted access and increased anxiety about public spaces. The new guidelines imposed on healthcare settings may have led to longer waiting times, restricted clinic hours, and limited patient capacity, which further dissuaded caregivers from seeking vaccination services for their children. These factors likely contributed to lower vaccination rates at Site D compared to other sites.” (lines 419- 425)

Self-Reported Data Limitations:

In addition, we have incorporated a limitation related to the self-reported nature of the data. The revised version reads: “Another limitation is the self-reported nature of the data, which may be affected by recall bias and social desirability bias, as caregivers might not accurately remember past vaccination events or may report more positive vaccine confidence to align with social expectations.” (lines 426- 429)

Future Research Directions: The suggestions for future research are reasonable. To make them more actionable, provide some initial steps or potential research questions for the proposed mixed - methods studies or larger cross - sectional analyses. For example, in a mixed - methods study, what specific qualitative data could be collected to complement the quantitative findings?

RESPONSE: Thank you for your thoughtful suggestions regarding future research. We agree that incorporating mixed-methods studies or larger cross-sectional analyses could provide valuable insights. To make these directions more actionable, we update the original sentence from: “Future research should aim to address these limitations by conducting larger, more diverse cross-sectional studies to improve the representativeness of findings. Mixed-methods studies combining quantitative and qualitative approaches could also provide deeper insights into the cultural, socioeconomic, and psychological barriers to HPV vaccination.” to “Future research should aim to address these limitations by conducting larger, more diverse cross-sectional studies to examine how these factors influence vaccination decisions across regions and communities. Mixed-methods studies could explore cultural, socioeconomic, and psychological barriers to HPV vaccination, using caregiver interviews to explore the reasons for vaccination decisions, and delays in vaccination.” (lines 465- 469)

Conclusion

Summary of Findings: The conclusion should more clearly summarize the key findings in a concise manner. List the most important factors influencing HPV vaccine uptake in a bullet - point format or a short, numbered list for easy reference.

RESPONSE: Thank you for your suggestion to improve the clarity and conciseness of the conclusion. In response to your feedback, we have revised the summary of findings to present the key factors in a clear, bulleted format for easier reference.

Original Conclusion:

“Our study identifies key factors influencing HPV vaccine uptake among adolescents, notably the effectiveness of the pay-it-forward intervention, caregivers’ knowledge of HPV vaccines, intentions to delay vaccination, caregiver occupation, and prior vaccine refusal.”

Revised Conclusion:

“Our study identifies the following key factors influencing HPV vaccine uptake among adolescents:

1. Effectiveness of the pay-it-forward intervention – Higher vaccination rates were observed in the pay-it-forward arm compared to the standard-of-care arm.
2. Caregivers’ knowledge of HPV vaccines – Understanding vaccine safety and effectiveness is crucial for promoting vaccination.
3. Intentions to delay vaccination – Caregivers who intended to delay vaccination were less likely to vaccinate their daughters.
4. Caregiver occupation – Caregivers' employment status influenced their decision to vaccinate.
5. Prior vaccine refusal – Caregivers who previously refused the vaccine were less likely to vaccinate.” （line 434-445）

Recommendations: The recommendations have been made more specific, but they could be further operationalized. For example, when suggesting financial subsidies for low - income families, provide some estimates of the amount of subsidy needed, or how it could be implemented in different regions of China. Also, describe in more detail how local health workers can be involved in fostering community engagement, such as the types of training they may need.

RESPONSE: Thank you for your valuable suggestion to operationalize our recommendations further. In response to your feedback, we have revised the recommendations section to include specific estimates for financial subsidies and a more detailed description of how local health workers can be involved in fostering community engagement.

Original Recommendation: “These findings highlight the importance of tailored health education initiatives that address specific knowledge gaps and misconceptions, particularly for caregivers in underserved communities. Providing financial subsidies remains a critical strategy to alleviate economic barriers, especially for low-income families. Furthermore, fostering community involvement through local health workers can strengthen trust and engagement among caregivers.”

Revised Recommendation: “These findings highlight the need for tailored health education to address specific knowledge gaps and misconceptions, especially among caregivers in underserved communities. Programs should focus on dispelling myths about the HPV vaccine, emphasizing its safety and effectiveness, and fostering community engagement through local health workers. These workers should be trained in vaccine knowledge, effective communication, and cultural sensitivity to organize outreach activities that build trust and increase vaccine uptake. Financial subsidies are essential to reduce economic barriers for low-income families. For example, a 45 USD subsidy, based on the cost of a domestic 2v-HPV vaccine, could encourage early initiation of vaccination and be implemented through government programs or local health insurance schemes to ensure equitable access.” (line 446- 456)

Overall Structure and Clarity

Consistency: Check for consistency in the use of terms throughout the manuscript. For example, ensure that "caregivers" and "parents" are used consistently, and if there are differences, clarify them.

RESPONSE: Thank you for your thoughtful suggestion. In this study, we use the term "caregivers" to refer to adults who play a role in making decisions or assisting with HPV vaccination for adolescent girls. This term is intentionally broader than "parents" and includes individuals who might not be the biological parents but are still responsible for the adolescent's well-being and health decisions, such as guardians, grandparents, or other family members. While some of the literature may use the term "parents" specifically to describe those responsible for care and decision-making, our use of "caregivers" encompasses a wider range of caregiving roles.

To address your concern, we can add a clarification in the methods section to explicitly define "caregivers" and highlight the distinction between this term and "parents." This will ensure that readers understand our choice of terminology and how it applies to the participants in our study. We have updated the methods section with the following clarification:" For the purposes of this study, the term 'caregivers' is used to refer to adults who are responsible for making healthcare decisions for adolescents, including but not limited to biological parents. This broader term encompasses individuals such as guardians, grandparents, or other family members involved in caregiving roles."(line 115-119)

Readability: Some sentences are quite long and complex, especially in the discussion section. Break them into shorter, more digestible sentences to improve readability. Use more subheadings in the discussion section to further organize the content, even if not strictly in a sub - chapter format. This would help readers quickly locate and understand different aspects of the discussion.

RESPONSE:

Thank you for your valuable feedback on the readability and organization of the discussion section. We have carefully revised the section to improve both clarity and flow. Specifically, we broke down long and complex sentences into shorter, more digestible ones, which we believe enhances the overall readability.

In addition, we have added subheadings to better organize the content and guide readers through the discussion. These subheadings will allow readers to easily locate and understand different aspects of the study. The subheadings introduced are as follows:

Impact of the Pay-it-Forward Intervention (line 324)

Barriers to HPV Vaccine Uptake (line 360)

Global Relevance and Application of the Findings (line 397)

These changes were made to improve the structure of the discussion section, helping readers better follow key points and arguments. While these revisions do not alter the content or framework of the paper, we believe they significantly enhance the manuscript's clarity and readability.

We sincerely appreciate the time and effort the Editors and Reviewers have dedicated to providing insightful comments and suggestions. We hope that these revisions meet your expectations and are happy to receive any further feedback.

Once again, thank you for your invaluable feedback and guidance.

Sincerely,

Dr. Jing Li

Department of Epidemiology and Statistics, West China School of Public Health and West China Fourth Hospital, Sichuan University, Chengdu, China, 610041

Email: [lijingwcph@scu.edu.cn](mailto:lijingwcph@scu.edu.cn)
