# Supplementary material for: Factors affecting caregivers’ HPV vaccination decisions for adolescent girls: A secondary analysis of a Chinese RCT
Source: PLoS One. 2025 Jun 17;20(6):e0324260. doi: 10.1371/journal.pone.0324260 (PMC12173375; doi:10.1371/journal.pone.0324260)
Supplement: Attachment — Submitted filename: Response to Reviewers-third.docx [file pone.0324260.s007.docx]

Apr 21^st^,2025

Dear Dr. Emily Chenette,

We are pleased to resubmit our manuscript, previously titled “Factors Influencing Caregivers’ Action to Vaccinate Adolescent Girls Against HPV: Secondary Analysis of a Randomized Controlled Trial in China”, and now retitled “Factors Affecting Caregivers’ HPV Vaccination Decisions for Adolescent Girls: A Secondary Analysis of a Chinese RCT”, for consideration for publication in *PLOS One*. The manuscript ID for the submission was PONE-D-24-51311R2.

We are grateful for the constructive and insightful comments from the reviewers, which have helped us improve the quality and clarity of our manuscript. We have carefully reviewed our reference list to ensure that it is complete and accurate. In response, we have carefully revised the manuscript and have included a detailed point-by-point response below. All revisions are marked in red in the manuscript for your convenience.

Reviewer #5:

1. Methods: The subsidy amount is mentioned, but not the total cost of the vaccine. This is necessary context for the reader.

RESPONSE: Thank you for this valuable suggestion. To address your comment, we have added the following information to the manuscript: :“The total cost for a full three-dose HPV vaccine series in the local market ranged from approximately CNY 990 to CNY 3,900 (USD 139–546), depending on the type and manufacturer.” (Lines 160-162)

2. Results: The percentage of caregivers who paid it forward is not mentioned in the results. My assumption, from seeing the pilot publication, is that this result will be shared in the main RCT publication. Please reference where this can be found, as it leaves the reader wondering.

RESPONSE: We appreciate your thoughtful observation. The proportion of caregivers who paid it forward will indeed be detailed in the main RCT manuscript, which is currently under peer review. As these data are not yet publicly available, we are unable to cite them at this time. We will ensure to update the reference in future versions. Thank you for your understanding.

3. Results: Am I correct to assume that “Have you ever heard of HPV”, “Annual family income”, and “Is there anyone around who has been vaccinated against HPV” were not <0.2 after adjusting for age and gender as they were not included in the multivariable analysis? Please mention why these were not included in the multivariable analysis.

RESPONSE: Thank you for this important clarification request. The variables “Have you ever heard of HPV,” “Annual family income,” and “Is there anyone around who has been vaccinated against HPV” had p-values less than 0.2 after adjusting for age and gender, and therefore, were included in the multivariable model. However, they were later excluded through backward stepwise logistic regression, which retained only the most significant predictors of HPV vaccine uptake. This has been clarified in the manuscript (Lines 312–313).

4. Results & Discussion: If available, please provide the number of participants approached in screening, how many were sent the questionnaire, and how many were finally included for this analysis. This is vital to understand potential bias in the results and contextualise findings, such as a higher vaccine uptake in the non-intervention group being attributed to active engagement. In addition, if high drop-out rates were found, please reflect on their impact on the results in the limitations.

RESPONSE: Thank you for highlighting this important point. A total of 321 participants were approached during screening and all 321 were sent the questionnaire. The questionnaire completion rate was 100% in both the pay-it-forward and standard-of-care arms. Thus, all participants were included in the final analysis. This information has been added to the manuscript (Lines 267–269). As no participant dropped out or declined to respond, we believe potential bias due to non-response or attrition is minimal.

Reviewer #6:

Line 217: “were more likely to vaccinate their adolescents” → consider rephrasing to "were more likely to have their daughters vaccinated" for smoother flow.

RESPONSE: Thank you for this helpful stylistic suggestion. We have revised the phrase accordingly to “were more likely to have their daughters vaccinated” at both Line 286 and Line 300 in the manuscript for improved clarity and flow.

Line 294: “ensuring access to vaccination services for participants willing to consider other formulations” could be made more concise.

RESPONSE: Thank you for pointing this out. We have revised the phrase to a more concise version: "ensuring access to vaccination services for participants willing to consider other formulations" to make it more concise. The revised version is now as follows: " ensuring access to vaccination for participants considering other formulations"(Lines 434-435)

We sincerely appreciate the time and effort that you and the reviewers have invested in reviewing our manuscript. We are confident that the revisions we have made address the concerns raised and significantly improve the manuscript. We hope it will now meet the journal’s requirements for publication.

Once again, thank you for your invaluable feedback.

Sincerely,

Dr. Jing Li

Department of Epidemiology and Statistics, West China School of Public Health and West China Fourth Hospital, Sichuan University, Chengdu, China, 610041

Email: [lijingwcph@scu.edu.cn](mailto:lijingwcph@scu.edu.cn)
